# Osteo-Compatibility of 3D Titanium Porous Coating Applied by Direct Energy Deposition (DED) for a Cementless Total Knee Arthroplasty Implant: In Vitro and In Vivo Study

**DOI:** 10.3390/jcm9020478

**Published:** 2020-02-09

**Authors:** Dong Jin Ryu, Hun Yeong Ban, Eui Yub Jung, Chung-Hee Sonn, Da Hee Hong, Shakra Ahmad, Bomi Gweon, Dohyung Lim, Joon Ho Wang

**Affiliations:** 1 Department of Orthopedic Surgery, Samsung Medical Center, Sungkyunkwan University School of Medicine, Seoul 06351, Korea; mdryu24@naver.com; 2 Department of Mechanical Engineering, Sejong University, Seoul 05006, Korea; ban2946@sejong.ac.kr (H.Y.B.); gweonbomi@gmail.com (B.G.); 3 Department of Orthopedic Surgery, National Medical Center, Seoul 04564, Korea; sinceric2864@gmail.com; 4 Samsung Biomedical Research Institute, Samsung Medical Center, Sungkyunkwan University School of Medicine, Seoul 06351, Korea; chsonn@hanmail.net (C.-H.S.); dahee1994@hanmail.net (D.H.H.); shakrach98@gmail.com (S.A.); 5 Department of Health Sciences and Technology, SAIHST, Sungkyunkwan University, Seoul 06351, Korea; 6 Department of Medical Device Management and Research, SAIHST, Sungkyunkwan University, Seoul 06351, Korea

**Keywords:** direct energy deposition, titanium porous coating, osseointegration, osteoblast, cementless TKA, 3D printing

## Abstract

Direct energy deposition (DED) technology has gained increasing attention as a new implant surface technology that replicates the porous structure of natural bones facilitating osteoblast colonization and bone ingrowth. However, concerns have arisen over osteolysis or chronic inflammation that could be caused by Cobalt-chrome (CoCr) alloy and Titanium (Ti) nanoparticles produced during the fabrication process. Here, we evaluated whether a DED Ti-coated on CoCr alloy could improve osteoblast colonization and osseointegration in vitro and in vivo without causing any significant side effects. Three types of implant CoCr surfaces (smooth, sand-blasted and DED Ti-coated) were tested and compared. Three cell proliferation markers and six inflammatory cytokine markers were measured using SaOS2 osteoblast cells. Subsequently, X-ray and bone histomorphometric analyses were performed after implantation into rabbit femur. There were no differences between the DED group and positive control in cytokine assays. However, in the 5-bromo-2′-deoxyuridine (BrdU) assay the DED group exhibited even higher values than the positive control. For bone histomorphometry, DED was significantly superior within the 1000 µm bone area. The results suggest that DED Ti-coated metal printing does not affect the osteoblast viability or impair osseointegration in vitro and in vivo. Thus, this technology is biocompatible for coating the surfaces of cementless total knee arthroplasty (TKA) implants.

## 1. Introduction

Total knee arthroplasty (TKA) is a common procedure in orthopedics for treating end-stage osteoarthritis of the knee joint. Although cemented TKA is reported to have excellent long-term survival [1], cement has poor resistance to shear force and is associated with the risk of aseptic loosening of the tibial component [2]. With more interest in biologic fixation, there is a growing interest in cementless fixation. Despite advances in technology, the current cementless TKA implants are still limited by the lack of appropriate cell adhesion and osseointegration, leading to reduced lifespan of the implant [2,3,4]. These are hurdles for widespread use of cementless TKA.

The success of cementless TKA depends on biological (osseointegration between the implant and bone) and mechanical (rigid primary fixation, high stress shield) stability [2]. In the presence of micro-motion, an inflammatory reaction and fibrous tissue may develop rather than the ideal bony ingrowth, leading to aseptic loosening and mechanical failure of TKA [5]. Thus, an important aspect of TKA implant integration is the enhancement of functional osteoblast activity at the tissue-implant interface without the intervention of any fibrous tissue [3]. Recent efforts have highlighted the importance of surface topography of materials and composites to better mimic the surface features of natural bone with biologically active porous coatings such as hydroxyapatite [2,3,6].

Various surface coating methods have been developed to accelerate the osseointegration process and improve biocompatibility [7,8]. One such method is the direct energy deposition (DED) technique which has significant advantages, in terms of manufacturing, mechanical strength, and biocompatibility. DED is a 3D metal printing technique that can be used to fabricate porous structures similar to that of human cancellous bone while maintaining the mechanical strength [9,10,11]. Another key advantage of the DED technique is the capability to fabricate an implant using two different materials by spraying the metal powder onto the base formed with different types of metal [10,11,12]. By using two different materials, we can design an implant that takes advantage of both materials. For example, the implant formed with porous Ti coating on CoCr alloy base is likely to produce less wear debris than the one formed with Ti solely [13]. 

In addition, the porous structure of DED is shown to be beneficial in cell adhesion and mechanical stability [12,14]. Recently, however, concerns have arisen over the periprosthetic osteolysis or chronic inflammation that could be caused by CoCr and Ti nanoparticles produced during the fabrication process [15,16,17,18]. To address this concern, a previous study had evaluated biocompatibility of DED Ti-coated on CoCr alloy [12]. However, to assess the functional benefits in terms of osseointegration of the DED Ti-coated surface considering the actual TKA implant position and motion of joint in vivo model, further study is essential. Here, we have tested cellular viability and measured inflammatory cytokines of osteoblasts grown on DED surfaces to evaluate the biocompatibility and safety of DED to the bone. Subsequently, to further evaluate whether a DED Ti-coated on CoCr alloy improves osseointegration compared to a pure CoCr alloy, osteoblast colonization and bone ingrowth were examined in vitro (SAOS2 cells) and in vivo (rabbits).

## 2. Materials and Methods

### 2.1. Preparation of Specimens

**Figure 1:**
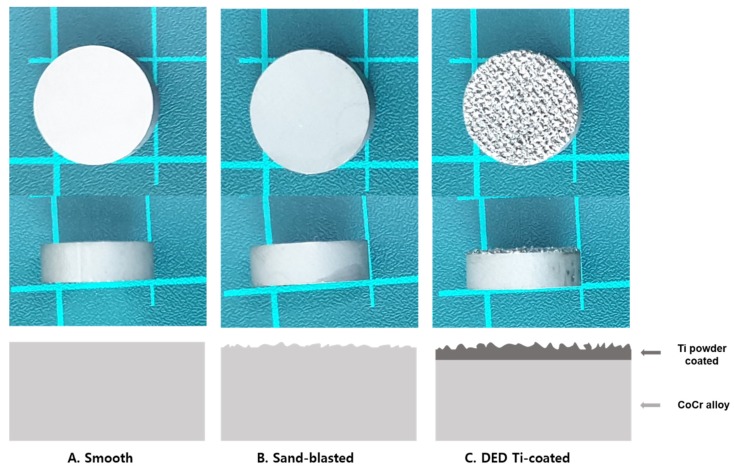
Photo viewed from the front and lateral side of each specimen and a schematic diagram used for experiments **A**: smooth, **B**: sand-blasted, **C**: direct energy deposition (DED)-Ti coated. (Black color: Cobalt Chrome alloy, Red color: Ti powder coating).

We prepared a porous layer of pure Ti (grade 2, ASTM F1580) on a CoCr substrate following the DED coating process. The porous structure was then manufactured using a 3D computer-assisted design program that created a sufficient fixation force by matching the material to the properties of the cancellous bone. The surface was irradiated using the following parameters: laser power: 100 W; scan speed: 1.5 m/min; power delivery rate: 2.2 g/min by following a pre-programmed path along a grid, which formed a melted pool. Next, metal powders were sprayed and laminated onto the surface to create a coating layer (average thickness: 500 µm) [10,19]. In addition, smooth (polished) and sand-blasted specimens were prepared for comparison (Figure 1).

### 2.2. Sample Preparation and Test Groups

We studied three types of implant surfaces (smooth, sand-blasted, and DED) in vitro and in vivo to compare the osteoblast activity and osseointegration using a Cell Counting Kit-8 (CCK-8), Alkaline Phosphatase (ALP), BrdU cell proliferation, and inflammatory cytokine assays, and in vivo X-ray and bone histomorphometric analyses. To set the baseline, a positive control, a cell-only group was included in the in vitro experiments. For in vitro study, on the basis of the data obtained from our previous study [20], a sample size calculation (*α* = 0.05, *β* = 0.2) was conducted in terms of the mean and standard deviation of optical density (OD) value of CCK-8 assay using G power 3.1 [21]. Six specimens in each in vitro study are needed as the minimum requirement to ensure 80% power. For the in vivo study, on the basis of the data in the other previous similar study [12], a sample size was calculated in terms of the mean and standard deviation of bone-to-implant contact using G power 3.1. A sample size of five specimens in each group was calculated as the minimum requirement to ensure 80% power for detecting differences. Thus, three types of specimen (diameter: 14.6 mm; height: 3 mm fitted for 24 well plate) were manufactured for in vitro study (*n*  =  90). As each in vitro experiment had been repeated six times, 30 specimens (6 repetition x 5 types of in vitro experiment) were required for each specimen type. Therefore, (1) smooth (*n*  =  30), (2) sand-blasted (*n* = 30), and (3) DED Ti-coated (*n*  =  30) were manufactured. Additionally, two types of specimen discs (diameter: 6 mm; thickness: 3 mm) were manufactured for the in vivo study (*n*  =  10); these were (1) smooth (*n*  =  5), (2) DED Ti-coated (*n*  =  5). 

### 2.3. In Vitro Preparation

All cell culture biologics were purchased from Gibco BRL (Grand Island, NY, USA) and HyClone (GE Healthcare Life Sciences Korea, Seoul, KOREA). The CCK-8 Kit for cell viability assay was purchased from Dojindo Molecular Technologies (Rockville, MD, USA) and the ALP Assay Kit was from Abcam (Cambridge, UK), the BrdU cell proliferation assay kit was from Cell Signaling Technology (Danvers, MA, USA) and the LEGENDplex^TM^ kit for cytokine assay was from BioLegend (San Diego, CA, USA).

#### 2.3.1. CCK-8 Viability Analysis of Cultured Osteoblast

Human Osteosarcoma cells (SaOS2; obtained from the Korean cell line bank, Seoul, KOREA) were seeded at a low (3 × 10^3^ cells/mL) and high concentration (1.2 × 10^4^ cells/mL) on the implant surface in a 24-well. The cells were incubated in a CO_2_ incubator at 37 °C for 1, 3, 5 or 7 days in RPMI Medium 1640 (Gibco-BRL; Thermo Fisher Scientific, Inc., Waltham, MA, USA) supplemented with 10% fetal bovine serum (FBS) and 1% Penicillin-Streptomycin. After incubation, CCK-8 solution (60 µL) was added in each well and incubated for 3 h in a CO_2_ incubator at 37 °C, following which, 100 µL from each well was taken to a 96-well plate. The absorbance was measured by Spectra MAX (MAX 190) microplate reader at 450 nm wavelength according to the manufacturer’s instructions.

#### 2.3.2. Alkaline Phosphatase (ALP) Activity Assay

SaOS2 Cells were seeded on implants and control wells in a 24-well plate with the same condition as described in 2.3.1. After 3 days of incubation, both of supernatant and lysate were collected and centrifuged at 3600 rpm (revolutions per minute) at 4 °C for 15 min. Subsequently, an ALP assay kit was used according to the kit manual. The absorbance was measured by microplate reader at 405 nm wavelength on Spectra MAX (MAX 190) microplate reader [3].

#### 2.3.3. BrdU Cell Proliferation Assay

To detect osteoblast cell proliferation, we used BrdU assay. When cells are cultured with labeling media which contains 5-bromo-2′-deoxyuridine (BrdU), this nucleotide analog (pyrimidine) becomes incorporated into replicating DNA in place of thymidine. Subsequently, immunodetection of BrdU using specific monoclonal antibodies allows labeling of cells in S phase of the cell cycle which is a direct indication of cell proliferation [22,23]. SaOS2 Cells were seeded on implants and control wells in a 24-well plate with the same condition as mentioned above. After 3 days of incubation, a BrdU cell proliferation assay kit was used according to the manufacturer’s instructions. The absorbance was measured at a wavelength range of 450–550 nm on a Spectra MAX (MAX 190) microplate reader.

#### 2.3.4. Inflammatory Multiplex Cytokine Assay

After 3 days of incubation (as detailed in Section 2.3.1), the supernatant from each well was collected for the cytokine assay. Reagents and standards were prepared according to the manual provided with the kit [24]. When the samples were ready for flow cytometry, FACS fluorescence activated cell sorting (FACS, BD Bioscience, Franklin Lakes, NJ, USA) was used for flow cytometry analysis. The results were further analyzed by LEGENDplex v.8.0 software (BioLegend, San Diego, CA, USA). This assay allows the simultaneous quantification of 13 different cytokines. We focused on 6 cytokines, namely TNF-α, MCP-1, IL-1β, IL-6, IL-12p70, and GM-CSF (Granulocyte-macrophage colony-stimulating factor) [3,25]. 

### 2.4. In Vivo Preparation

Twenty 36-week-old female New Zealand white rabbits with an average weight of 3.8–4 kg were used in this study. Animals were individually housed in a light, temperature and humidity (temperature: 23 ± 2 °C, humidity: 60 ± 10%) controlled environment and provided with food and water freely under a 12-h light cycle. Animal management and surgical procedures were performed in accordance with the National Institutes of Health guide for the care and use of laboratory animals and the standards issued by the Ethics Committee on Animal experimentation at Samsung Medical Center. (SMC 2018-0713-002) The experiment animals were divided into two groups, smooth and DED Ti-coated (DED), and the animals were euthanized at 12 weeks after implantation. There was one case of expiring at 2 days after implantation surgery in the smooth group. It was judged to have expired due to acute stress. As a result, one more rabbit was allocated to the smooth group, and a total of five smooth and five DED Ti-coated subjects were obtained.

#### 2.4.1. Surgical Procedure

**Figure 2:**
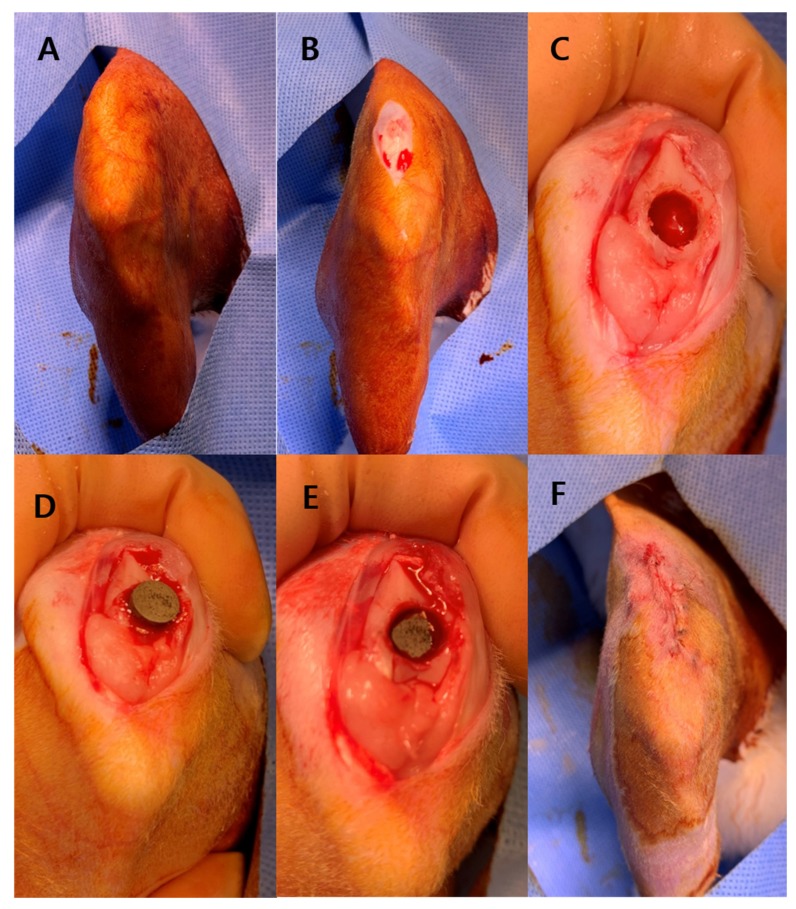
The surgical procedure for implanting specimens into the rabbit trochlear groove. **A:** The right knee of each rabbit was shaved and sterilized with povidone-iodine. **B**: In the supine position, the right legs were incised longitudinally from 2 cm above the knee joint to 1.5 cm below the knee joint. **C**: A hole was created with a 6-mm drill bit in the proximal side of trochlear groove. **D**: A specimen was placed in the hole with the surface towards the cancellous bone. **E**: The specimen was gently impacted to contact with the cancellous bone. **F**: Reduction of the patella and repairing of the incised structure with Vicryl 2–0.

General anesthesia was induced by an intramuscular injection of Ketamine (700 μL/Kg) and Xylazine hydrochloride (200 μL/Kg). The right knee of each rabbit was shaved and sterilized with povidone-iodine (Figure 2A). In the supine position, the right legs were incised longitudinally from 2 cm above the knee joint to 1.5 cm below the knee joint (Figure 2B). 

From the superomedial side of the patella, the vastus medialis muscle was incised through the medial side patella and patella tendon to the proximal end of the tibia tuberosity. This exposed the trochlear groove and the condyle of femur with sliding of the patella to the lateral side. A hole in the proximal side of trochlear groove was created with a 6-mm drill bit, taking care to ensure that the hole was gently reamed (Figure 2C). To prevent thermal injuries around the bone and soft tissue, normal saline was sprayed during reaming. An experimental specimen was placed in the hole of trochlear groove with the surface towards the cancellous bone (Figure 2D). We gently impacted the specimen to contact with cancellous bone (Figure 2E). Patella reduction and repair of the incised structure with Vicryl 2–0 was performed (Figure 2F). Finally, the wound was disinfected by povidone-iodine.

#### 2.4.2. Postoperative Care and Sacrifice

**Figure 3:**
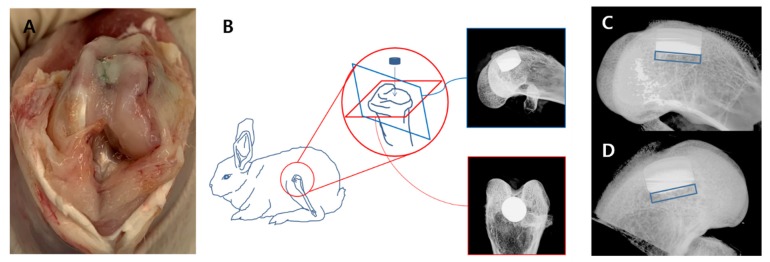
**A**: After euthanizing, the right side of the distal femur was harvested. **B**: Axial and lateral images were obtained using micro X-ray. **C**, **D**: Osteolysis was defined as a low-density area visible within a 1-mm area from bone-implant contact surface (lateral-view).

After surgery, the rabbits were administered 0.6 mL/kg of Cefazoline (Chongkundang, Seoul, Korea) and 1.8 mL/kg Ketoprofen (UNIBIO tech, Seoul, Korea) intramuscularly three times daily for three days. For behavioral observation, the rabbits were allowed to act freely within the cage after surgery. Subsequently, the rabbits were euthanized at 12 weeks after implantation. We injected Ketamine (700 μL/Kg) and Xylazine hydrochloriide (400 μL/Kg) intramuscularly followed by an intravenous injection of potassium chloride. Then, the right side of distal femur was harvested (Figure 3.A), and the specimens were fixed in 10% neutral buffered formalin (Sigma-Aldrich Corp. St. Louis, MO, USA) for two weeks.

#### 2.4.3. X-ray

Axial and lateral images were acquired using Inveon CT (Siemens, Munich, Germany; Voltage: 70 kV, Current: 400 µA, Exposure: 400 ms) (Figure 3B). Osteolysis was defined as a low-density area visible within a 1-mm area from bone-implant contact surface in a lateral-view image (Figure 3C,D).

#### 2.4.4. Histologic Slide Manufacturing and Staining

After the X-ray analysis, specimens were cleaned with distilled water, and decalcification was performed using ethylenediaminetetraacetic acid solution (pH 9.0) (Zytomed systems GmbH, Berlin, Germany) for 5 weeks. After confirming the removal of calcium, the specimens were embedded in paraffin and sectioned to a thickness of 50 µm with a hard tissue slicer (Struers, Willich, Germany) [26]. Sections were stained with hematoxylin and eosin (H&E; Sigma-Aldrich, St. Louis, MO, USA) and Masson’s trichrome (MT; Sigma-Aldrich, St. Louis, MO, USA) stain to visualize contact surface and osseointegration. General specimen imaging and histomorphometric analyses at ×40 were conducted by a professionally trained, blinded investigator (D.H. Hong). 

#### 2.4.5. Bone Histomorphometry

**Figure 4:**
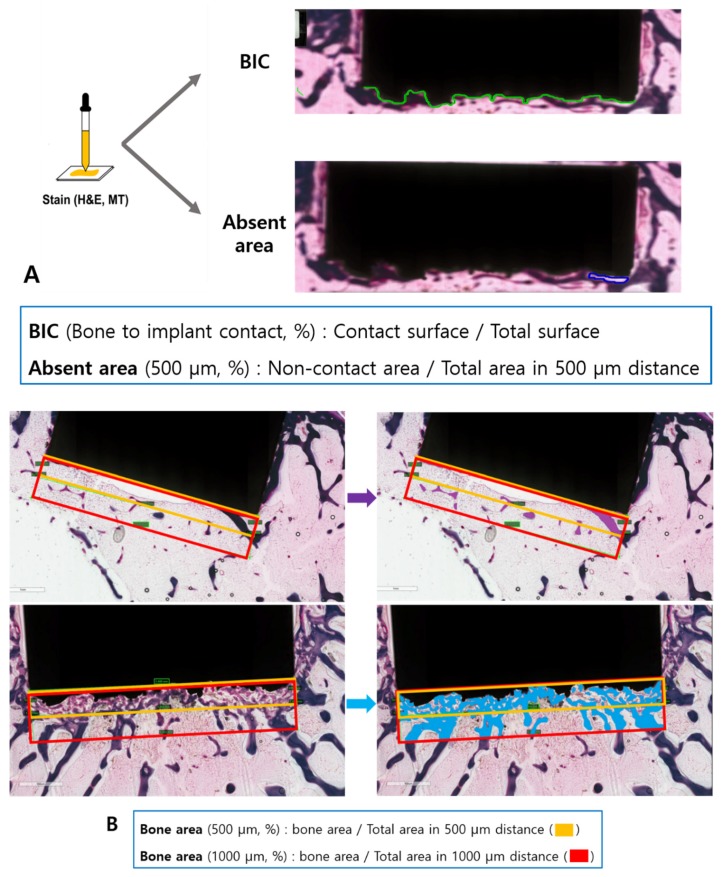
After the staining procedures, we performed bone histomorphometry using four methods. **A**: (1) Bone-to-implant contact (BIC): determination of the percentage of direct contact surface between the mineralized bone and the surface of the CoCr alloy; (2) absent area: determination of the percentage of non-contact area between the total area in a 500 µm region. **B**: (3) bone area (500 µm): determination of the percentage of new bone formation and neovascularization area between the total area in a 500 µm region; 4) bone area (1000 µm): determination of the percentage of new bone formation and neovascularization area between the total area in a 1000 µm region.

Light microscopy images were obtained using ×12.5, and ×100 objectives (BX51, Olympus, Tokyo, Japan). Images were captured using a digital camera attached to the microscope (CC-12, Soft Imaging system GmbH, Munster, Germany) [27]. The specimens from each implant were analyzed by (1) bone-to-implant contact (BIC): determining the percentage of direct contact surface between mineralized bone and the CoCr alloy surface; (2) absent area: determining the percentage of non-contact area between the total area in a 500-µm region (Figure 4A); (3) bone area (500 µm): the percentage of new bone formation and neovascularization area between the total area in a 500-µm region; (4) bone area (1000 µm): the percentage of new bone formation and neovascularization area between the total area in 1000-µm region (Figure 4B) [27,28,29].

### 2.5. Statistical Analysis

A Kruskal-Wallis test was used to compare differences between the experimental groups. For comparison between each experimental group, multiple Mann-Whitney *U* tests were used and adjusted using the Benjamini & Hochberg’s method [30]. All analyses were performed using SPSS^®^ 25.0 software (SPSS, Chicago, IL, USA). A *p* value < 0.05 was considered significant.

## 3. Result

### 3.1. In Vitro CCK-8 Assay

**Figure 5:**
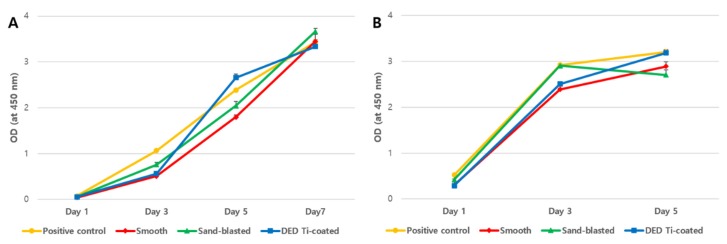
Results of CCK-8 assay for each specimen at the time flow using low (**A**: 3 × 10^3^ cells) and high (**B**: 1.2 × 10^4^ cells) concentrations of cells. In the DED group, unlike that in the other groups, the osteoblast viability increased rapidly between from day 3 to 5. (OD: optical density, DED: direct energy deposition).

At low concentrations of osteoblast (3 × 10^3^), all four experimental groups showed increasing viability over time, reaching their maximum value on day 7 (Figure 5A). In the DED group, unlike that in other groups, the osteoblast viability increased rapidly between from day three to day five. The high cell concentration (1.2 × 10^4^) experiment showed a pattern of reaching the maximum viability at day five with no significant difference being observed among the four experimental groups (Figure 5B).

### 3.2. In Vitro ALP Assay

**Figure 6:**
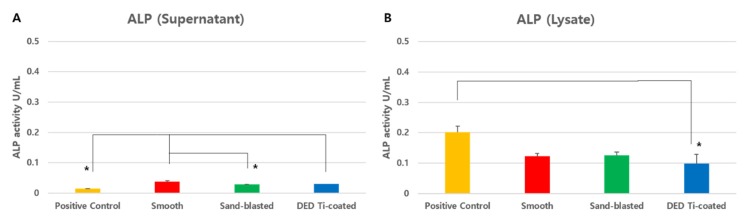
The result of alkaline phosphatase (ALP) activity assay from supernatant (**A**) and lysate (**B**). A: In the supernatant, there were significant differences among the four experimental groups (*p* = 0.0003). In the comparison between groups, the positive control group exhibited significantly lower ALP activity than the smooth and DED groups. B: In the lysate, ALP activity was lower in the DED group than in that in the positive control. (* *p* < 0.05). (ALP: alkaline phosphatase, DED: direct energy deposition).

ALP activity, measured only in the supernatant of the cell culture fluid, was significantly different between the four experimental groups (*p* = 0.0003). ALP activity was higher in the smooth (*p* = 0.0002) and DED (*p* = 0.0186) groups than that in the positive control, and ALP activity in the sand-blasted group was lower than that in the smooth group (*p* = 0.039, Figure 6A). The ALP level in cellular lysate was statistically significantly lower in the DED group than that in the positive control group (*p* = 0.0197, Figure 6B), but there was no significant difference between the other three specimens (*p* > 0.05).

### 3.3. In Vitro BrdU Cell Proliferation Assay

**Figure 7:**
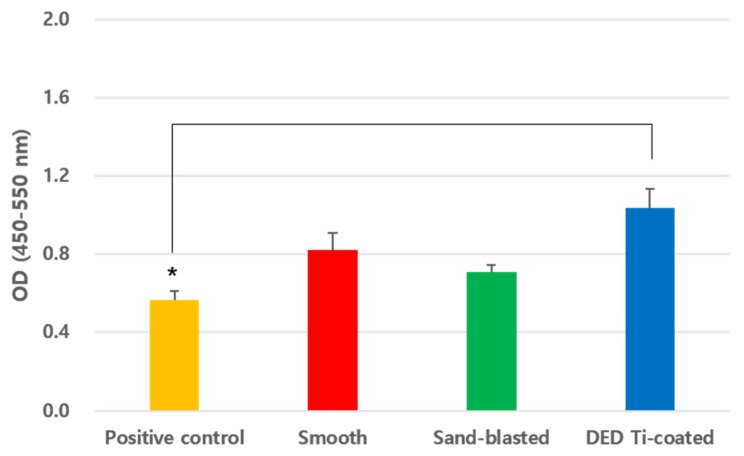
Result of BrdU cell proliferation assay showing that the DED group exhibited significantly higher value than the positive control (*p* = 0.0027). There were no significant differences between the between the smooth, sand-blasted, and DED Ti-coated specimens; however, the positive control showed a significant difference. (* *p* < 0.05). (BrdU: 5-bromo-2′-deoxyuridine).

In the BrdU assay measured on day three, the DED group exhibited significantly higher BrdU levels than that of the positive control (*p* = 0.0027, Figure 7). There were no significant differences between the three specimens, except for positive control.

### 3.4. In Vitro Inflammatory Cytokine Assay

**Figure 8:**
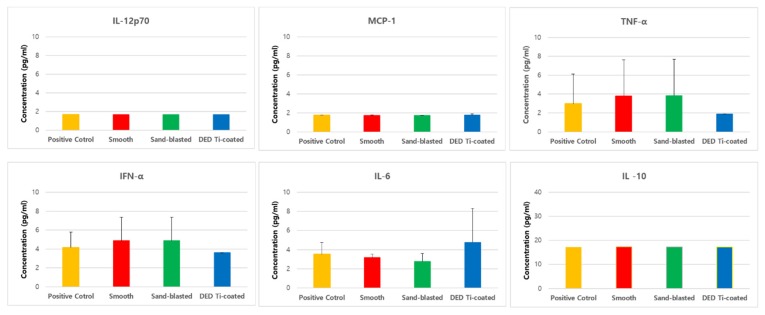
Results of the inflammatory cytokine multiplex assay showing that there were no significant differences among the four experimental groups with respect to cytokine production (all *p* > 0.05). (IL: interleukin, MCP: monocyte chemoattractant protein, TNF: tumor necrosis factor, IFN: interferon).

Levels of all of the six inflammatory cytokines (IL-12p70, MCP-1, TNF- α. IFN-α, IL-6, IL-10) produced by osteoblast were not significantly different between the test groups (Figure 8).

### 3.5. In Vivo X-ray

Neither smooth nor DED groups observed any definite osteolysis in the 1-mm margin of the bone-implant contact surface in X-rays taken at 12 weeks.

### 3.6. In Vivo Bone Histomorphometry

**Figure 9:**
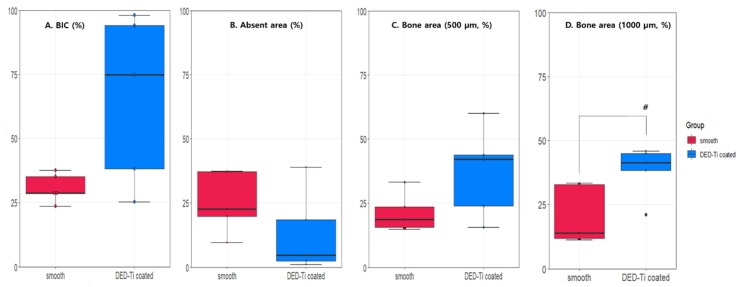
The results of bone histomorphometry. In **A**: BIC (*p* = 0.095), **B**: absent area (*p* = 0.222), and **C**: 500 µm bone area (*p* = 0.095) there were no significant differences between the smooth and DED groups. **D**: However, in the 1000 µm bone area, the DED group exhibited a significantly higher new bone formation than the smooth group (*p* = 0.0317). (# *p* < 0.05).

The BIC (smooth: 30.68 ± 5.59%, DED Ti-coated: 66.06 ± 32.87%, *p* = 0.095) and absent area (smooth: 25.27 ± 11.95 %, DED Ti-coated 13.05 ± 15.98%, *p* = 0.222) were not significantly different between the two groups (Figure 9A,B). Although the DED group was not statistically significant in the 500 µm bone area (smooth: 21.17 ± 7.55 %, DED Ti-coated 37.08 ± 17.52%, *p* = 0.095), in the 1000 µm bone area, the DED group had a statistically higher area than that of the smooth group (smooth: 20.67 ± 11.44 %, DED Ti-coated 38.26 ± 10.09%, *p* = 0.0317, Figure 9C,D).

## 4. Discussion

This study aimed to determine whether a DED Ti-coated on CoCr alloy is biocompatible in vitro and in vivo. An abnormal cytotoxic reaction was not identified in the CCK-8 viability assay and on the basis of inflammatory cytokine responses. In the CCK-8 assay with a low cell concentration, there was a non-significant trend toward reaching the maximum viability on day seven, and with a high cell concentration there was a trend toward reaching a maximum on day five, in all four experimental groups. There were no significant differences among all four test groups with respect to the ALP assay using supernatant; however, in experiments using the lysate, the DED group exhibited low ALP activity. There were no significant differences when only three types of specimens were compared.

In the BrdU assay, the DED group exhibited higher values than the positive control, suggesting that it results in high cell proliferation. Osteoblast proliferation is dictated by the surface properties of the material, such as chemical composition, roughness, curvotaxis, and surface energy [31,32,33,34,35]. Even if two surfaces are composed of the same materials, the porosity and convexity are known to have a critical effect, so it can be determined that the DED coating method with a porous structure induces osteoblast proliferation and osteogenesis. This may be related to the higher cell viability increase in the DED group between day three and five in low cell concentration CCK-8 assay. 

Osteolysis evaluated by X-ray was not observed in both the smooth and DED groups, but micro-CT analysis is necessary to make an accurate evaluation [36]. However, due to the metal interference effect of CoCr alloy and titanium, an accurate image of the bone-implant contact surface could not be obtained. Definite osteolysis can be confirmed in CT in actual clinical practice, but fine osteolysis of the surface is often difficult to identify due to interference effects [37]. This is a limitation in this study.

The ingrowth of bone tissue into pores is critical to obtain successful osseointegration for a porous coating implant. Although the optimal pore size required for bone ingrowth is still to be determined, the consensus is that it should be greater than 100–200 µm [38,39,40]. If the pore size is small (< 100 µm), the cells can span the pores directly by the elongation of the cell itself. For large pores (> 200 µm), cells cannot span directly across the pores and grow into the pores. Thus, early osseointegration can occur and high bone-implant contact surface area can be achieved [39]. Using DED technology, the characteristics of the surface can be controlled to create ideal porous surfaces with ideal pore size and maximum roughness [9,41]. In addition, adding an antibacterial and bioactive coating to the titanium surface increases the efficiency of osseointegration and prevents infection [42,43,44].

Our study has some limitations. First, our sample size was relatively small. In particular, the in vivo animal model had limitations due to experimental ethics. Second, the purpose of this study was to evaluate the osteoblast activity and osseointegration of DED specimens themselves, but further investigation is needed to compare them with other cementless TKA surface coating techniques, such as powder bed fusion (PBF), titanium plasma spray (TPS), and Hydroxyapatite (HA) coating. Third, further research is needed to compare the expression of bone formation markers (Ex. COL1A2, BMP-2, BMP-7) through immunohistochemistry staining [45]. Although the sections are technically difficult to evaluate due to their thickness, it is possible to quantitatively evaluate osteogenesis and assess the pattern of cytokine around the implant. Fourth, we needed to evaluate the correlations among cell number, surface adhesion, activity, and cell proliferation at each time point. This would allow the accurate interpretation of each measured values.

## 5. Conclusions

Titanium porous coating on the CoCr alloy using the 3D-DED metal printing technique does not affect osteoblast viability and does not impair osseointegration both in vitro and in vivo. This technology is therefore biocompatible for use in the surface coating of cementless TKA implants. Although the difference between two groups (smooth and DED Ti-coated) was not statistically significant, the BIC value was measured to be higher (smooth: 30.68%, DED Ti-coated: 66.06%). In addition, considering that the bone area is significantly higher in the 1000 µm area (smooth: 20.67%, DED Ti-coated: 38.26%), DED Ti-coating could be highly beneficial for osseointegration in clinic.
